# More or less equal? Trends in horizontal equity in mental health care utilization in Stockholm county, Sweden (2006–2022). *Repeated survey-registry linked studies*

**DOI:** 10.1186/s12939-025-02453-y

**Published:** 2025-04-08

**Authors:** Joseph Junior Muwonge, Beata Jablonska, Christina Dalman, Bo Burström, Maria Rosaria Galanti, Anna-Clara Hollander

**Affiliations:** 1 Department of Global Public Health, Karolinska Institute, Stockholm, Sweden; 2 Centre for Epidemiology and Community Medicine, Stockholm Health Care Services, Region Stockholm, Stockholm, Sweden

**Keywords:** Concentration index, Migrants, Mental disorders, Healthcare reforms, Covid-19

## Abstract

**Background:**

Horizontal equity is defined as equal care for equal needs, regardless of socioeconomic factors. This study investigated trends in horizontal equity in mental health care (MHC) utilization in Sweden from 2006 to 2022. Monitoring equity provides valuable information for healthcare system governance (e.g., planning and resource allocation) necessary for ensuring equitable provision of services.

**Methods:**

A total of 81,650 Stockholm residents aged 18–64, who participated in the Hälsa Stockholm surveys of 2006, 2010, 2014 or 2021, were analysed. Their subsequent use of MHC (primary, in- and outpatient specialized care, and psychotropic medication) within six months after survey response was collected from registries between 2006 and 2022. Concentration index (CI) and need-standardized CI (Horizontal inequity index, HI), summative measures of inequalities, were used in this study. HI was estimated using self-reported psychological distress (measured with the General health questionnaire 12 in 2006–2014 and Kessler 6 in 2021) as the primary need indicator, with general health status and long-term limiting illness as additional need indicators. Equivalized disposable household income was used as the ranking variable, while education status, migration status, age, and sex were included as non-need variables that we controlled for in the analyses.

**Results:**

Lower-income individuals used MHC services more than their higher-income counterparts with comparable levels of psychological distress. These “pro-poor” inequities in the probability of MHC use increased from HI = -0.057 [95% Confidence Limits, CL: -0.079, -0.034] in 2006/2007 to HI = -0.130 [95% CL: -0.159, -0.102] in 2014/2015. By 2021/2022, the “pro-poor” inequities had decreased (HI = -0.034 [95% CL: -0.06, -0.009]), partly due to an increase in MHC use among higher-income groups but a decrease in the lowest income group. Standardizing for additional need indicators reduced the “pro-poor” inequities but maintained the observed trends. Among non-Nordic migrants, “pro-rich” inequities fell between 2006/2007 and 2014/2015 but rose in 2021/2022, with significant “pro-rich” inequities among non-European migrants in 2021/2022 (HI = 0.100 [95% CL: 0.024, 0.176]). Among patients in outpatient services, “pro-poor” inequities in visit frequency decreased over time (2006–2022).

**Conclusion:**

We observed increasingly higher probability of MHC use among lower-income individuals than their higher-income peers with similar (measured) needs from 2006 to 2015. However, during the pandemic (2021/2022), potential access problems led to diminishing of “pro-poor” inequities in the total sample, and to “pro-rich” inequities among non-Nordic migrants. The Covid-19 disruption to the healthcare system—such as restrictions on in-person visits and the rapid transition to digital healthcare services—along with its impact on care-seeking, may explain the trend shifts.

**Supplementary Information:**

The online version contains supplementary material available at 10.1186/s12939-025-02453-y.

## Introduction

Mental disorders are common and they directly affect about 30% of the global population at least once during their lifetime [1], the majority of whom are individuals with lower socioeconomic status [2, 3]. In countries with strong universal healthcare coverage (with minimized financial barriers), including Sweden, evidence suggests that lower socioeconomic groups utilize mental health care (MHC) services more frequently than higher socioeconomic groups [4–12], in line with the policies on equitable and need-based care. However, exceptions exist: non-Nordic migrants are less likely to use services compared with Swedish-born individuals [13, 14], and lower socioeconomic groups are less likely to use services like psychotherapy and counselling compared with higher socioeconomic groups [11, 12]. Moreover, whether the overall higher utilization is proportionate to their greater needs is not clear given that only few studies account for differences in MHC needs [8–12].

### Current evidence on trends in inequities in mental healthcare use

Exploring trends in equity in MHC use remains largely unstudied, yet it is crucial for informing policy and governance (e.g., for planning and resource allocation), particularly in providing oversight on the healthcare system’s role in ensuring equitable use of healthcare services [15]. A registry-based study in Sweden (1994–2011) found increasing income-related differences in inpatient care, with higher usage in poorer individuals, but this study did not account for MHC needs [16]. In Belgium, a study found a stable higher use of psychotropic medication by people with shorter education from 2001 to 2018, even when adjusting for self-reported psychological distress and long-term illness [17]. Studies in England [18] and Canada [19] both found a stable 5-year link between living in poor neighborhoods and hospitalization (adjusting for several factors including prevalence of severe mental illness in neighborhoods). An Australian study, using data from 2009 to 2017 and accounting for self-rated health status and psychological distress, found no significant inequities in MHC use [20]. Most research has focused on inpatient care, which handles severe cases, and which may reflect poor use of primary and specialized outpatient services [21]. To our knowledge, no study has examined trends in inequities in outpatient service use, where most cases (specifically milder cases) are treated, and income-related inequities may follow a different pattern.

### Healthcare system and societal trends over time

Several factors and events in the past 20 years may have impacted access to, and the equitable utilization of services in Sweden. First, the “market-oriented” primary care reforms in Stockholm County (2008) and across Sweden (2010) aimed to create competition among healthcare providers and improve access to care by allowing private providers to establish primary care facilities (provided they met defined criteria), and giving patients the freedom to choose their provider [22, 23]. Evaluations of the reform’s impact on access to primary care and equity found mixed results, though there were indications of geographical inequity (tendency to establish facilities in more affluent areas) [23–27].

Second, while the overall prevalence of mental health problems seems stable, it has increased in lower socioeconomic groups [16, 28]. Meanwhile limited service availability, indicated by long wait times and a slight reduction in the density of mental health providers [29, 30], suggests that the healthcare system may not be keeping up with the growing needs of this group [31].

Third, changing demographics, particularly the growing immigrant population, pose unique challenges for the healthcare system in delivering need-based MHC. Like other European countries, Sweden received a large number of refugees in 2014–2015 [32]. Refugees, in particular, have a higher prevalence of mental disorders than the host population [33] due to traumatic experiences before, during, and after migration [34, 35]. For the healthcare system, this patient group presents unique experiences, cultural backgrounds, and language barriers, which may require longer consultations and specialized care. At the same time, the general immigrant population, which now makes up ∼ 20% of the population in Sweden (2023 figures [36]), utilizes fewer MHC services than the host population [13], meaning this group’s utilization patterns may influence overall inequity trends.

Fourth, the rising economic inequalities in Sweden [37] may negatively impact mental health and access to MHC services [38]. Evidence suggests that countries/areas with higher economic inequalities are associated with a higher prevalence of mental disorders [39–41], probably due to a larger marginalized population who are at higher risk of mental disorders [2]. Moreover, given that this marginalized group (at the poorest end of the rank) may experience financial barriers to MHC services [42], widening economic inequalities could lead to more unmet needs in the population over time.

Lastly, the Covid-19 pandemic (2020–2022) not only impacted care-seeking behaviour [43] but also disrupted the healthcare system, as access to MHC services was limited or delayed due to the prioritization of Covid-19 patients [44]. Physical visits were restricted, and online services were promoted to improve access, leading to a rapid increase in their utilization [45]. For instance, subscribers of the *Alltid Öppet* app, a healthcare app owned by Region Stockholm, increased from 40,000 in early 2020 to over two million by 2022 [46]. A pre-pandemic study (data from 2018) found that individuals with higher socioeconomic status were more likely to use online primary care services than their counterparts with lower socioeconomic status [47].

Taken together, these health system changes, societal trends, and rising mental health problems highlight the need to monitor changes in access to MHC services and changes in equity in MHC use over time [48]. We examined trends in income-related equity in MHC use from 2006 to 2022, covering the period before the primary care reforms (2008) and during the pandemic.

## Methods

### Setting

In Sweden, need-based healthcare is a key goal of the Swedish Healthcare Act [15] and is central to many policies and reforms [49]. This study was conducted in Stockholm County. With about 2.5 million residents (Statistics Sweden, 2024), it is the most populous of the 21 counties in Sweden. MHC is primarily provided by region-financed private and public facilities. Privately funded care is almost negligible, with only 1% of the total healthcare expenditure in Sweden financed by private health insurance [50]. Adults’ access to MHC is based on assessed needs, beginning with primary care, and if needed, specialized care (in- and outpatient care), and acute cases are managed at emergency departments. Adults pay subsidized user-fees for services, but there is a cap on out-of-pocket expenditure within a 12-month period in outpatient care (starting from the initial visit) and for prescribed medication (from the first purchase) [51]. The system is designed to reduce financial barriers to care, mitigate the economic impact of healthcare use, and ensure need-based access to services. Nevertheless, cost remains a concern, as some individuals limit or refrain from seeking healthcare due to financial constraints [42].

### Materials/data sources

This study used data from the Stockholm Public Health Cohort (SPHC) database, a survey-registry linked database owned by Region Stockholm. It includes a cross-sectional sample of residents selected using stratified random sampling from Stockholm’s municipalities and districts who participated in one of the Hälsa Stockholm surveys, conducted every four years. For this study, we used data from the survey waves conducted in 2006 (response rate: 61%), 2010 (55.6%), 2014 (42.3%) and 2021 (48.2%). The Region did not conduct the Hälsa Stockholm survey in 2018. As is common in surveys, respondents of the Hälsa Stockholm surveys are more likely to be female, older, married, Swedish-born, and of higher socioeconomic status than non-responders or general population [52, 53].

Healthcare records in the SPHC come from the regional healthcare database “VAL-databaserna”, covering primary care (optimal coverage from 2014), specialized outpatient and inpatient care, and prescribed drugs (2016–2022). These records comprise of care received in public and region-financed private facilities in Stockholm County [54]. Data on prescribed medication for the years before 2016 was collected from the national Prescribed Drugs Registry, managed by the National Board of Health and Welfare. Supplementary primary care data was also collected from old primary care files (KON) for the 2006 and 2010 waves to identify individuals missing from the main outpatient registry [55]. In addition, a significant proportion of MHC use in primary care is captured by using records on psychotropic medication (∼ 58% of those who used primary care in 2021/2022, when we had good coverage of primary care records, had also collected psychotropic medication).

Information on educational level, household income, and country of birth, was collected (in the same year as the surveys) from the Longitudinal integration database for Health Insurance and Labor Market Studies (LISA) and the Total Population Registry (TPR) at Statistics Sweden (SCB).

### Study design

This study employed a survey-registry linked cohort design to examine equity in MHC use among adults living in Stockholm County. The study measured MHC use over a six-month period from the date of participants’ survey responses regarding psychological distress. A six-month follow-up period was selected to capture use of MHC close to the period of distress.

### Study population

**Fig. 1:**
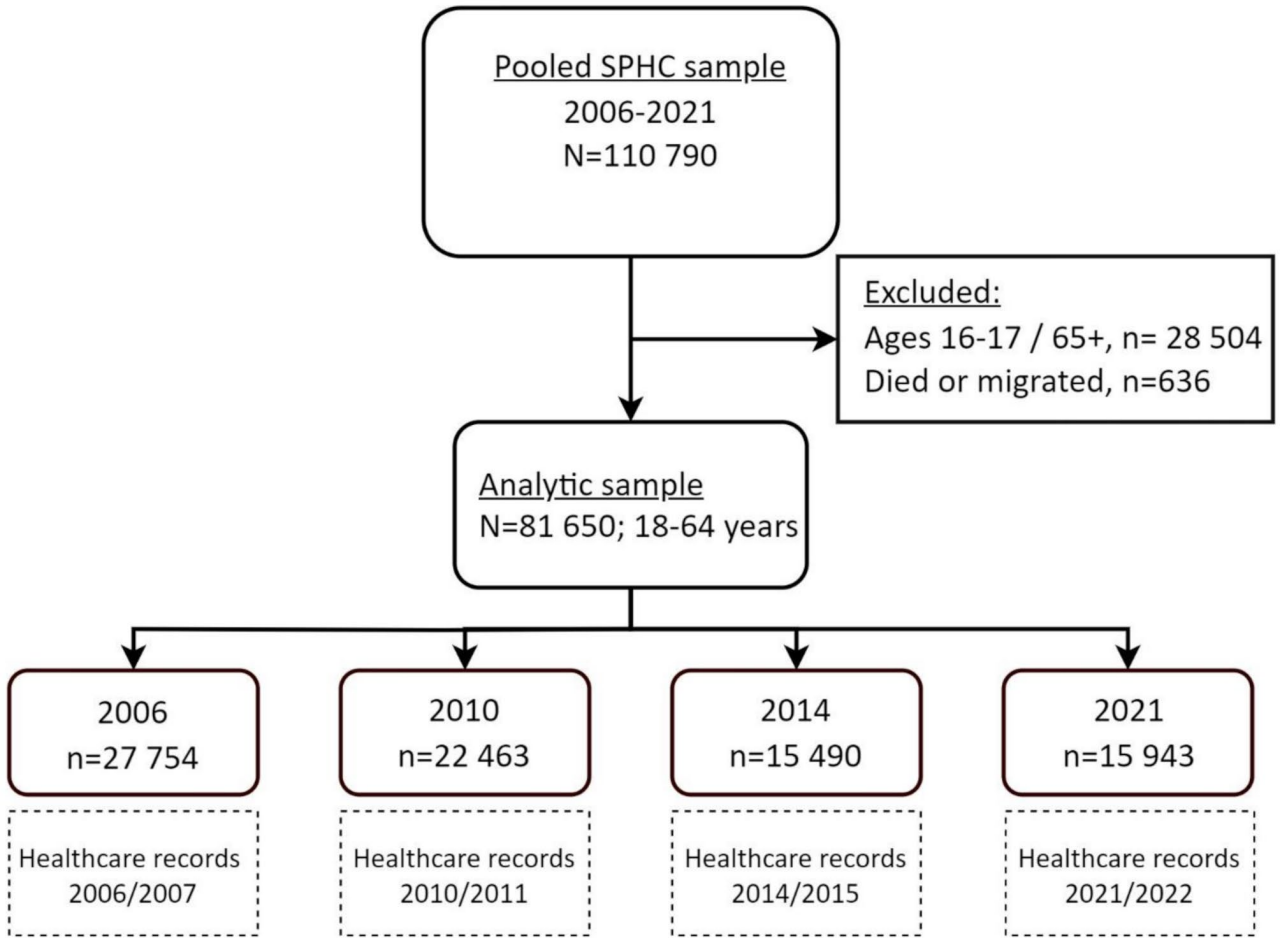
Derivation of the analytic sample. Individuals participated in one of the Stockholm Public health surveys “Hälsa Stockholm” between 2006 and 2021. Their healthcare records were collected from registries 6 months from survey-response between 2006/2007 and 2021/2022

As shown in Fig. 1, a total sample of 110,790 individuals, aged 16 years and older, participated in the *Hälsa Stockholm* 2006–2021. After the exclusion of three groups: adolescents aged 16–17 years, individuals aged 65 and above, and those who had died or emigrated during the 6-month follow-up period, our final sample consisted of 81,650 adults (18–64 years).

### Variable definition

As done previously [56, 57], we used four sets of variables to estimate need-standardized MHC use and horizontal inequity index: (1) the outcome variable, (2) ranking variable, (3) need variables and (4) non-need variables.

#### Outcome variable

MHC use was identified if a participant had a recorded psychiatric diagnosis or met a mental health professional during a visit or collected psychotropic medication *(see appendix for ICD-10 and ATC codes)*. We studied both (i) use of MHC at least once (probability) and (ii) frequency of utilization (how often) because we assume that the decision to seek MHC and the decision to engage in ongoing care—reflected in how often services are used—may be influenced by different factors [58]:

i. MHC use at least once in primary or specialized outpatient care (including online care), collection of prescribed medication, or hospitalization during the follow-up period.
ii. Number of primary and specialized outpatient visits conditional on at least one visit in primary or specialized outpatient care during the follow-up period.

#### Ranking variable

Income rank was based on the equivalized disposable household income. Equivalized disposable household income is calculated by SCB for each household/year and is weighted for household size and composition. A small random number was added to the income variable to remove ties before ranking. The weighted income rank was then used to estimate concentration indices and horizontal inequity indices. However, for purposes of descriptive analyses e.g., predicted probabilities, we categorized household income into quintiles (5 groups).

#### Need variable(s)

Our primary need indicator was self-reported psychological distress and was measured with the 12-item general health questionnaire (GHQ-12) during 2006–2014 and the 6-item Kessler (K6) psychological distress scale which replaced the former in 2021. Both the GHQ-12 and K6 are validated instruments used to screen for psychological distress (non-specific mental disorders) in population-based surveys [59, 60]. They measure how often or how much symptoms have affected a person’s functionality in the past few weeks (GHQ-12) or month (K6) [59, 61]. However, they differ in length, response options and reference period. Additionally, two of the three previous studies comparing the criterion validity of GHQ-12 and K6 found that K6 performs better in accurately screening for mental health conditions [62–64].

Based on previous studies including a K6 validation study, a score of 5–12 identifies individuals with moderate distress, while a score ≥ 13 identifies those with severe distress [60, 65]. The corresponding scores on the GHQ-12 were obtained from a conversion table produced by a study that equated scores on the GHQ-12 and K6 using equipercentile methods [66]. Individuals were categorized into three groups based on their scores on the GHQ-12 or K6: score of 0 on the GHQ-12 or 0–4 on K6 as “no distress”, 1–7 on the GHQ-12 or 5–12 on K6 as “moderate distress”, and 8–12 on the GHQ-12 or 13–24 on K6 as “severe distress”.

Additional need indicators included the self-rated General Health Status and Long-Term Limiting illness. We considered these factors as additional indicators of MHC needs, given the presentation of somatic symptoms in patients with mental disorders [67, 68] and the established link between physical conditions and mental disorders [69–71]. The General Health Status was measured by the question, *“How do you rate your general health status?”* with responses 1*) very good*, *(2) good*, *(3) somewhat good*, *(4) poor*, *and (5) very poor*. Long-Term Limiting illness was measured using the question: *“Do you have any long-term sickness*, *discomfort following an accident*, *reduced physical function*, *or any other long-term health problem?”* with responses *(1) yes or (2) no*.

#### Non-need variables

Non-need variables are factors that on their own are not considered as indicators of need for services, but that may influence healthcare utilization [72]. In this study, we considered education level, migration status, age, and sex as non-need factors when estimating income-related inequities in MHC use. These could also be considered as confounders or moderators of the relationship between income and MHC use.

*Education level* was categorized into no/primary education, secondary, and post-secondary education. *Migration status* was collected already categorized as born in Sweden, other Nordic countries, Europe, and outside Europe. For stratified analyses, we primarily categorized migration status as Nordic-born (including Sweden) and non-Nordic born but also presented separate results for those born in Sweden and those born outside Europe (to understand observed patterns in the primary classification). *Age* was categorized into 18–29 and 30–64 years, and *sex* into men and women (recorded at birth).

### Statistical analysis

#### Procedure – Concentration index

To measure inequalities, we used the Concentration index (CI), a method that provides a summary measure of inequalities in a health variable (here, MHC use). Concentration indices measure how MHC use is distributed in a population ranked from poorest to richest. The Standard CI is bounded between − 1 and + 1, with higher scores indicating higher inequality. The negative (positive) value indicates that MHC use is more concentrated in lower-income individuals (higher-income individuals), and zero indicates equality.

Need-standardized CI or referred to as Horizontal Inequity Index (HI Index) measures the principle of horizontal equity. Similar to CIs, a negative value indicates that MHC use is more concentrated among lower-income individuals than would be expected given measured need (“pro-poor”), a positive value indicates inequity favoring higher-income individuals (“pro-rich”), and zero indicates horizontal equity i.e., MHC use is distributed according to measured need in the population [72, 73].

#### MHC use at least once

We used the indirect standardization method to estimate need-standardized probabilities of MHC use as suggested by Wagstaff and colleagues for microdata [72]. Indirect standardization assumes that after standardizing for observable need variables and controlling for observable non-need variables, any remaining inequalities, such as those by income, are not justified by differences in (measured) need [72]. The procedure entails firstly estimating need-predicted MHC use for each individual using the probit model (due to the binary nature of the outcome variable) while controlling for non-need variables (at their sample means) [72]. That is, the expected MHC use by an individual, on average, if they were to be treated the same way the system treats others with similar (measured) needs.

$$Y_i^X = \widehat \alpha + \widehat \beta {X_i} + \widehat \delta \mathop {{Z_1}}\limits^ - $$

Where $\:{Y}_{i}^{X}\:$is the need-predicted MHC use for an individual i, $\:{X}_{i}$ is(are) the need variable(s), and $\:\stackrel{-}{{Z}_{1}}$ the sample means of the non-need variables.

Secondly, need-standardized MHC use is then estimated for each individual by subtracting the need-predicted MHC use from the observed MHC use and adding back the mean of the need-predicted MHC use [72]. Need standardized MHC use ($\:{Y}_{i}^{ST}$) is therefore equal to observed MHC use ($\:{Y}_{i}$) minus need-predicted MHC use ($\:{Y}_{i}^{X}$) plus mean of need-predicted MHC use ($\:\stackrel{-}{{Y}^{X}}$) [72].

$$Y_i^{ST} = {Y_i} - Y_i^X + {\bar Y^X}$$

To obtain concentration indices with robust standard errors, we used the “convenient regression approach” [72] with survey (calibrated) weights applied and with the Wagstaff normalization for binary outcomes [72]. The concentration index of the need-standardized MHC use ($\:{Y}_{i}^{ST}$) is then the estimate of horizontal inequity index i.e., measure of income-related inequities in MHC use, after standardizing for needs.

All analyses were run separately for each year. Additional analyses stratified by age-group, sex and migration status were run to test if observed income inequities in MHC use (both in terms of direction and magnitude) vary by age-group, sex, and migration status.

#### Frequency of visits

Need-standardized frequency of visits (count data) was estimated using zero-truncated negative binomial regression and then concentration indices and HI indices estimated using the ‘convenient regression approach’ [72], similar to the steps above (except for Wagstaff normalization). To minimize the effect of outliers on the estimate, we excluded 13 cases where patients had more than 60 outpatient visits during the 6-month follow-up period.

#### Sensitivity analyses and post hoc analyses

To account for the influence of prior MHC use on reported distress and follow-up visits, we measured inequities in incident MHC use, excluding those who used MHC six months before the survey. However, excluding previous users may bias the income-related inequity measurement if previous users are more likely to be from lower income groups.

Due to the poor coverage of primary care records before 2014, we conducted a separate analysis excluding primary care from the main outcome variable. We anticipated that if primary care facilities serving wealthier or poorer populations were more likely to report data (before 2014), it could bias our results on trends.

In addition, we have provided separate results by healthcare level and contact type (physical/digital) for 2014/2015 and 2021/2022, when primary care record coverage was optimal, as income-related inequities likely vary by healthcare level or type of contact [47].

For comparison purposes, we have presented odds ratios and rate ratios for the relationship between income and MHC use (based on traditional regression approaches). Odds ratios and Rate ratios comparing lower-income quintiles to the highest income quintile (reference category) displayed similar trends as those observed using horizontal inequity indices for income inequities in MHC use (see Fig. S1-S2 & Table S1-S2 in the appendix).

#### Handling of missing values

Complete case analysis was performed due to a low proportion of missing: <0.1% for household income and 1% for psychological distress (ranging from 0.6% in 2014 to 1.7% in 2021).

All analyses were performed in STATA version 18.

## Results

Table 1 summarizes the characteristics of the 81,650 individuals who participated in the four survey waves from 2006 to 2021. The mean age of participants slightly increased over time. While the proportion of male participants was mostly stable, it rose significantly in 2021. The percentage of participants with post-secondary education increased from 43.7% in 2006 to 57.5% in 2021. The mean equivalized disposable household income per year increased from about 233,332 Swedish crowns in 2006 to 442,476 in 2021. Additionally, the proportion of non-Nordic migrants increased steadily, with European migrants increasing from 5.2 to 7.0% and non-European migrants from 8.9 to 14.1% by 2021 (Table 1).

**Table 1:** Characteristics of the study sample across survey waves, *unweighted proportions*

	Total	2006	2010	2014	2021	*P*-value^a^
	81,650 (100%)	27,754 (100%)	22,463 (100%)	15,490 (100%)	15,943 (100%)	
**Age**, **mean (std)**	43.05 (12.95)	42.61 (12.98)	42.81 (13.12)	43.45 (12.79)	43.77 (12.77)	
**Sex**, ***N*(%)**						0.0131
Men	36,631 (44.9%)	12,472 (44.9%)	9980 (44.4%)	6861 (44.3%)	7318 (45.9%)	
Women	45,019 (55.1%)	15,292 (55.1%)	12,483 (55.6%)	8629 (55.7%)	8625 (54.1%)	
**Education**						< 0.0001
Primary, ≤ 9 years	9068 (11.1%)	3692 (13.3%)	2678 (11.9%)	1390 (9.0%)	1308 (8.2%)	
secondary, 10–12 years	31,874 (39.0%)	11,820 (42.6%)	9243 (41.2%)	5514 (35.6%)	5297 (33.2%)	
Post-secondary, ≥ 13 years	40,250 (49.3%)	12,124 (43.7%)	10,463 (46.6%)	8494 (54.8%)	9169 (57.5%)	
Missing	458 (0.56%)	118 (0.43%)	79 (0.35%)	92 (0.59%)	169 (1.1%)	
**Migration status**^**c**^, ***N*(%)**						< 0.0001
Sweden	65,418 (80.1%)	22,521 (81.2%)	18,202 (81.0%)	12,462 (80.5%)	12,233 (76.7%)	
Nordic, other	2984 (3.7%)	1294 (4.7%)	879 (3.9%)	473 (3.1%)	338 (2.1%)	
Europe	4893 (6.0%)	1454 (5.2%)	1327 (5.9%)	992 (6.4%)	1120 (7.0%)	
Outside Europe	8346 (10.2%)	2479 (8.9%)	2055 (9.2%)	1561 (10.1%)	2251 (14.1%)	
**Household income**, **M (SD)**; *’000 Swedish crowns*	311.9 (483.5)	233.3 (296.4)	290.2 (385.0)	349.9 (331.3)	442.5 (836.2)	< 0.0001b
**Need indicators**						
Psychological distress (≥ 3 on the GHQ-12 or ≥ 8 on Kessler 6)	17,655 (21.6%)	5396 (19.4%)	4631 (20.6%)	3836 (24.8%)	3792 (23.8%)	< 0.0001
Poor/very poor rated general health status	3482 (4.3%)	1403 (5.1%)	873 (3.9%)	522 (3.4%)	684 (4.3%)	< 0.0001
Presence of long-term limiting illness/health problem	22,873 (28.0%)	7965 (28.7%)	6097 (27.1%)	4354 (28.1%)	4457 (28.0%)	< 0.0001

a-*P*-value was obtained from chi-square tests of differences across period

b-*P*-value based on ANOVA tests of differences in means in the four year-periods. Significant increases in mean household income were also observed in income adjusted for inflation (based on 2021 prices)

c-Migration status defined according to country of birth

### Income-related differences in need indicators

**Fig. 2:**
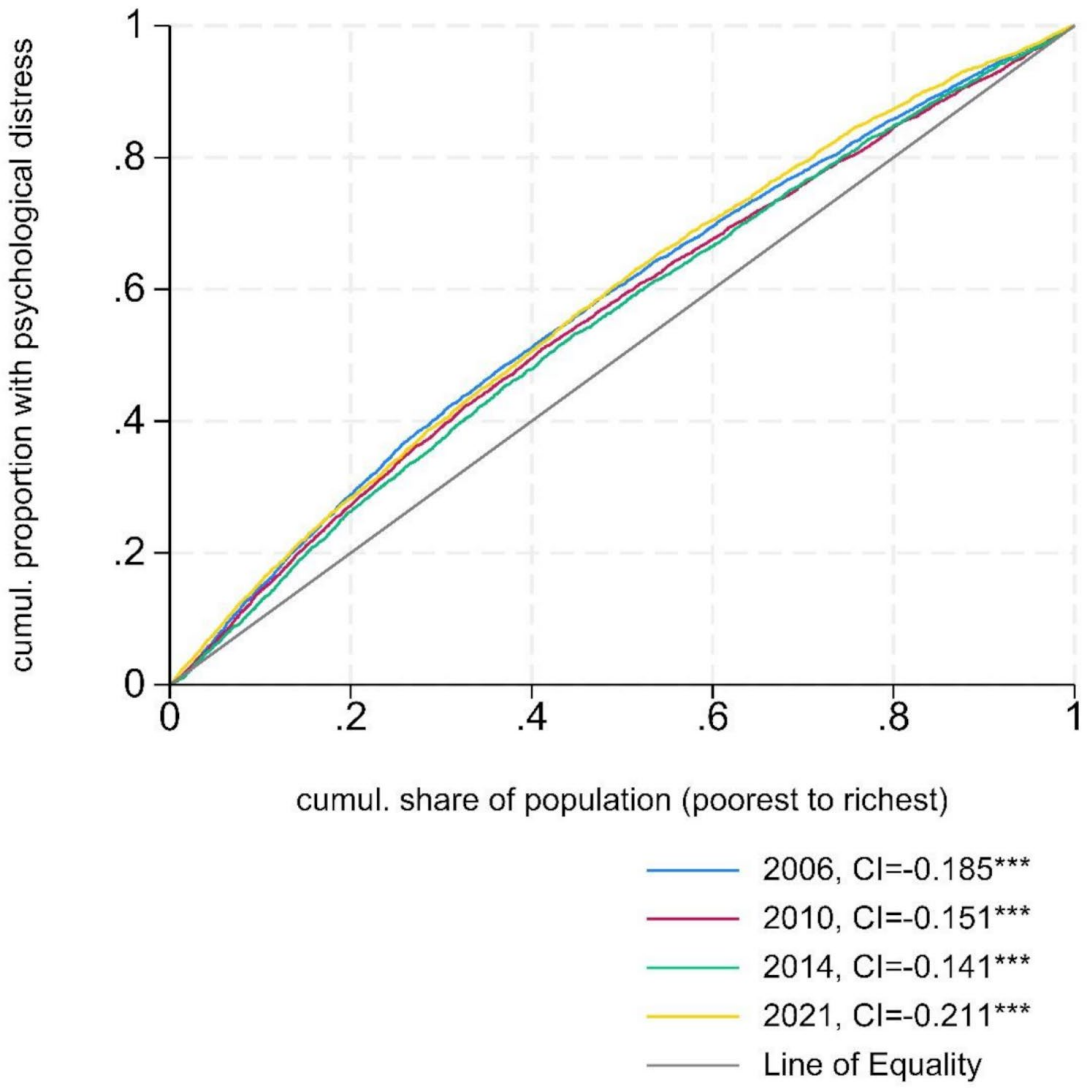
Concentration Curves showing the income-related inequalities in the probability of psychological distress. Distress defined as scoring ≥ 3 on the GHQ-12 (2006–2014) or ≥ 8 on the Kessler 6 (2021). *** - *P*-value < 0.0001. Curves above the Line of Equality indicate higher concentration of distress in individuals with lower income, “pro-poor inequalities”. The closer the curve is to the Line of Equality, the more equal the distribution of psychological distress among individuals with varying income

The prevalence of psychological distress, defined as scoring ≥ 3 on the GHQ-12 or ≥ 8 on Kessler 6, was consistently higher among lower-income groups. Figure 2 shows that the concentration curves for all four waves lie above the diagonal line, indicating that distress was higher among individuals with lower income (2006–2021), with the highest income-related inequalities observed in 2021 (CI = -0.211 [95% Confidence Limits, CL: -0.235, -0.187]). Lower income individuals were also more likely to rate their general health as poor/very poor and to report having long-term limiting illness or health problems (see concentration curves in the appendix, Fig. S3-S4).

### Need-standardized income inequalities in MHC use

**Fig. 3:**
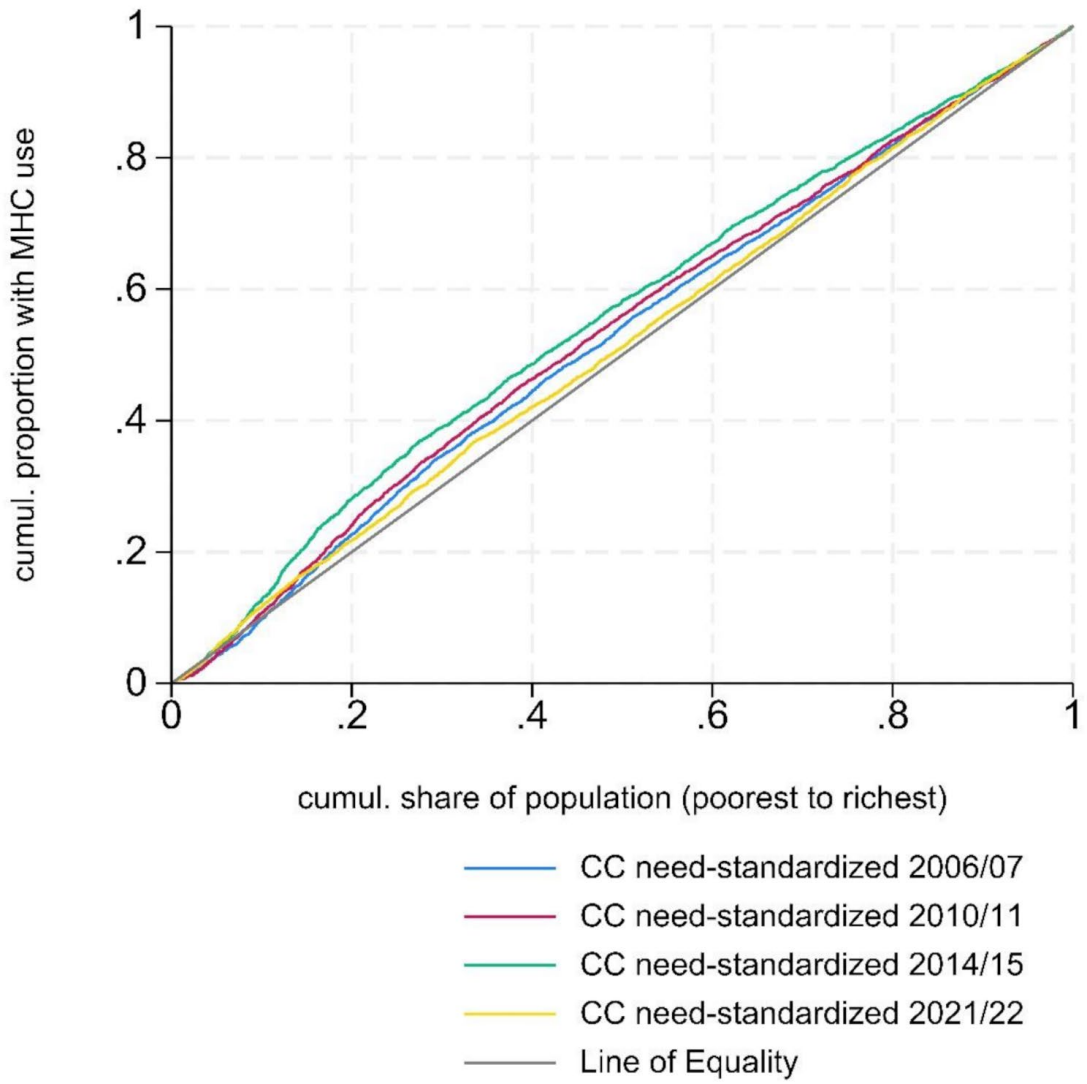
Concentration Curves (CC) showing the distribution of need-standardized MHC use across the income rank. Models standardized for psychological distress (as the only need-indicator: distress measured using the GHQ-12 in 2006–2014 and Kessler 6 in 2021) and controlled for education, sex, age-group and migration status. The closer the curve is to the Line of Equality, the more equal the distribution of need-standardized MHC use among individuals with varying income

Table 2 shows the observed, need-predicted, and need standardized probabilities of utilizing MHC services over a 6-month follow-up period across income quintiles, with need proxied by psychological distress. Lower-income groups consistently utilized MHC services more than the highest-income group (before and after need standardization). In 2006/2007, the (need-standardized) probability of MHC use was 15.9% for the lowest-income group and 12.5% for the highest-income group. By 2020/2021, these probabilities were 20.0% and 17.2%, respectively. The “pro-poor” inequities (higher utilization among lower-income individuals on the income rank), measured by the HI index, increased from HI = -0.057 [95% CL: -0.079, -0.034] in 2006/2007 to HI = -0.130 [95% CL: -0.159, -0.102] in 2014/2015. However, by 2021/2022, compared with the 2014/2015 period, the probability of MHC use had reduced in the lowest-income group while it increased in the higher-income groups, the HI index therefore reduced towards the null (0) with HI = -0.034 [95% CL: -0.06, -0.009]. As shown in Fig. 3, the concentration curve of need-standardized MHC use in 2021/2022 lies closest to the line of equality.

**Table 2:** Trends in the probability of MHC use across income quintiles. *Concentration index (before need-standardization) and horizontal inequity index (after need-standardized) shown for each year. Need proxied by psychological distress*

Household income, quintiles	2006/2007			2010/2011			2014/2015			2021/2022		
	Observed MHC use	Need-predicted MHC use	Need-standardized MHC use	Observed MHC use	Need-predicted MHC use	Need-standardized MHC use	Observed MHC use	Need-predicted MHC use	Need-standardized MHC use	Observed MHC use	Need-predicted MHC use	Need-standardized MHC use
Low	17.7%	15.1%	15.9%	19.2%	15.4%	17.7%	23.3%	16.9%	22.1%	23.1%	20.8%	20.0%
2	15.0%	13.4%	14.8%	15.4%	14.1%	15.3%	16.3%	15.9%	15.9%	19.6%	18.9%	18.3%
3	12.4%	12.8%	12.8%	12.3%	13.4%	12.9%	14.5%	15.1%	14.9%	17.6%	17.3%	18.2%
4	12.1%	12.4%	13.0%	12.3%	13.0%	13.1%	12.6%	14.8%	13.2%	17.6%	16.6%	18.9%
High	11.3%	12.0%	12.5%	11.9%	12.8%	12.8%	12.1%	14.4%	13.2%	14.2%	15.0%	17.2%
CI	-0.108***	-0.054***		-0.122***	-0.046***		-0.166***	-0.039***		-0.117***	-0.082***	
HI			-0.057***			-0.081***			-0.130***			-0.034*

Notes: Significance of Concentration indices: *p < 0.05; **p < 0.001; *** *p* < 0.0001; n.s – not significant; HI – Horizontal Inequity indices; Models controlled for education, sex, age-group and migration status

Distress measured using the GHQ-12 in the first three waves (2006–2014) and Kessler 6 in 2021

**Fig. 4:**
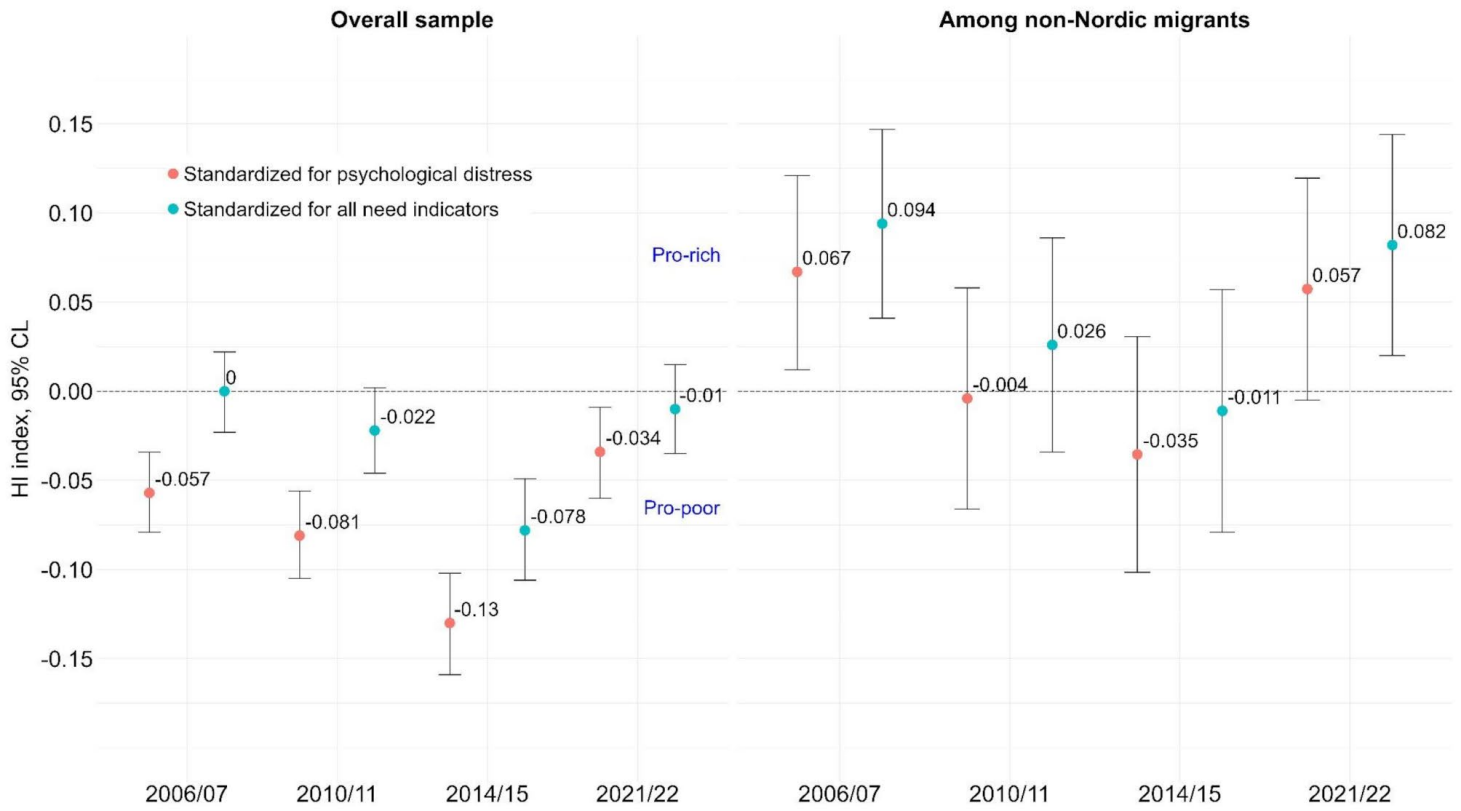
Horizontal inequity indices in MHC use over time, shown for the overall sample and among non-Nordic migrants. Negative values indicate higher MHC use in lower income individuals in comparison to those with higher income, “pro-poor inequities”. Point estimates and 95% confidence limits (CL) shown for models standardizing for psychological distress and models standardizing for all need indicators. Models controlled for education, sex, age-group and (migration status in the overall sample)

Standardizing for additional indicators of MHC needs using self-rated General Health Status and Long-Term Limiting illness reduced the observed “pro-poor” inequities to mostly non-significant levels, but the trends in inequities were similar to those observed above (see Fig. 4 and Table S3 in the appendix).

Trends in income-related inequities in MHC use by sex and age were similar to those observed in the total sample (see Table S3 in the appendix).

The analysis, stratified by migration status, revealed increasingly “pro-poor” trends in MHC use for all groups between 2006 and 2015. For Swedish- and Nordic-born individuals, the “pro-poor” inequities were attenuated but remained significant after standardizing for all need indicators. In 2021/2022, Swedish- and Nordic-born individuals still had significant “pro-poor” MHC use patterns. However, non-Nordic migrants—particularly non-European migrants—initially exhibited a “pro-rich” pattern (2006/2007), which then shifted to a “pro-poor” trend between 2010/2011 and 2014/2015. By 2021/2022, this pattern had reverted to “pro-rich” MHC use (non-European migrants: HI = 0.100 [95% CL: 0.024, 0.176]; Table S3 in the appendix).

### Need-standardized income inequalities in the frequency of outpatient visits (among patients)

Table 3 shows the distribution of outpatient visits (before and after need-standardization) over a 6-month follow-up period across income quintiles, with need proxied by psychological distress. Lower-income groups consistently visited outpatient services more frequently than higher-income groups. However, over time, there was a decrease in the average number of visits among lower-income groups, while there was an increase in the average number of visits among higher-income groups. Consequently, the “pro-poor” inequities reduced over time from − 0.108 [95% CL: -0.153, -0.063] in 2006/2007 to marginal levels, HI = -0.039 [95% CL: -0.074, -0.004] by 2021/2022. Similar results were found when standardizing for additional need indicators (see Table S5 in the appendix).

Analysis stratified by sex and age revealed mostly similar falling trends in “pro-poor” inequity over the study period. In addition, Swedish- or Nordic-born persons showed more pronouced “pro-poor” patterns in outpatient service use, but these inequities reduced over time. Separate analysis among non-Nordic migrants was not possible due to few cases (see Table S5 in the appendix).

### Results of the sensitivity analysis and posthoc analysis

Measuring inequities in incident MHC use (excluding individuals who used services prior to reporting distress) showed similar trends, but the “pro-poor” inequities were largely attenuated (see Table S4 in the appendix). The mean income (adjusted for inflation) in previous users was ∼ 325,871 SEK and in non-previous users it was ∼ 350,667 SEK.

Exclusively analysing MHC use based on complete datasets i.e., collected prescribed psychotropic medication and specialized MHC services yielded similar estimates and trends (see Table S4 in the appendix).

To understand the change in trends between 2014/2015 and 2021/2022, we examined utilization patterns by healthcare level and type of contact. Table S6 shows trends in the proportion who used MHC services by healthcare level or type of contact. In the first two waves (2006/2007 and 2010/2011), when primary care record coverage was poor, less than 2% had at least one visit to primary care for MHC use. With improved reporting, the proportion using primary care increased to 5.64% in 2014/2015 and 8.86% in 2021/2022. Need-standardized probabilities of MHC use in primary care increased for all income groups but increased more (larger % increase) for higher-income groups (see Table S7 in the appendix). Need-standardized probabilities of specialized MHC use, psychotropic medication, and physical (in-office) services declined for the lowest income groups but increased for higher-income groups in 2021/2022 compared with 2014/2015. Online service utilization increased for all groups in 2021/2022 (compared with 2014/2015) but the increase was much larger for higher-income groups.

Like in the main results above, “pro-poor” inequities in primary care use decreased from HI = -0.104 [95% CL: -0.149, -0.059] in 2014/2015 to HI = -0.032 [95% CL: -0.067, 0.004] in 2021/2022. “Pro-poor” inequities in specialized care use decreased from HI =-0.280 [95% CL: -0.323, -0.236] in 2014/2015 to HI = -0.178 [95% CL: -0.22, -0.136] (see Table S7 in the appendix).

Additionally, due to the change in distress measurement in the 2021 survey, we tested the robustness of the observed inequity trends in two ways: (i) by standardizing for need using only self-rated general health status and long-term limiting illness, which were consistently measured; and (ii) by using the average of the need-predicted concentration indices from the previous three waves, based on GHQ-12 measured distress (see Table 2), as the need-predicted CI for 2021/2022. The 2021/2022 HI index, based on approach 1, is -0.047 (using general health status and long-term limiting illness; see Table S8), and based on approach 2, it is -0.071 (using previous waves’ need-predicted CIs), compared with − 0.034 (using K6). Therefore, while the change in the distress measurement instrument affected the HI measurement, it did not alter the trend.

We tested the robustness of the observed trends in visit frequency by analyzing only specialized outpatient visits. We observed similar trends over time, i.e., declining “pro-poor” inequities, with non-significant inequities in both 2014/2015 and 2021/2022 (Table S9).

**Table 3:** Trends in the frequency of primary and specialized outpatient visits among patients. *Concentration index (before need-standardization) and horizontal inequity index (after need-standardized) shown for each year. Need proxied by psychological distress*

Household income, quintiles	2006/2007			2010/2011			2014/2015			2021/2022		
	Observed number of outpatient visits	Need-predicted number of outpatient visits	Need-standardized number of outpatient visits	Observed number of outpatient visits	Need-predicted number of outpatient visits	Need-standardized number of outpatient visits	Observed number of outpatient visits	Need-predicted number of outpatient visits	Need-standardized number of outpatient visits	Observed number of outpatient visits	Need-predicted number of outpatient visits	Need-standardized number of outpatient visits
Low	7.5	2.7	7.5	7.0	2.8	7.1	5.4	1.7	5.5	5.9	2.3	5.8
2	5.9	2.6	5.9	5.8	2.7	5.8	5.0	1.8	4.9	5.0	2.2	5.0
3	5.4	2.7	5.5	4.8	2.6	4.9	5.6	1.7	5.6	5.3	2.2	5.3
4	4.4	2.7	4.4	4.1	2.6	4.3	4.6	1.7	4.7	4.8	2.0	4.9
High	4.0	2.6	4.1	5.1	2.8	5.0	4.4	1.7	4.5	4.5	1.9	4.8
CI	-0.111***	-0.008 n.s		-0.106***	-0.010*		-0.052*	-0.010 n.s		-0.056**	-0.043***	
HI			-0.108***			-0.105***			-0.051*			-0.039*

Significance of Concentration indices: *p < 0.05; **p < 0.001; *** *p* < 0.0001; n.s – not significant; HI – Horizontal Inequity Index; Models controlled for education, sex, age-group and migration status

## Discussion

In this survey-registry linked study, lower-income individuals utilized MHC services more than higher-income individuals with the same levels of psychological distress. However, these “pro-poor” inequities were largely explained by their greater overall needs, as most inequities diminished after standardizing for additional need indicators. Between 2006 and 2015, inequities in the probability of MHC use became increasingly “pro-poor”, but by 2021/2022, these inequities diminished. This shift during Covid-19 was driven by “pro-rich” MHC use patterns especially among non-Nordic migrants. Among patients in outpatient services, “pro-poor” inequities in visit frequency diminished over time.

### Results in context

The higher utilization of MHC among individuals with lower income aligns with their higher need for these services. Standardizing for psychological distress, general health status, and long-term limiting illness eliminated most “pro-poor” inequities in MHC use but a “pro-poor” trend remained between 2006 and 2015.

This “pro-poor” trend in the probability of MHC use, even among non-Nordic migrants, is difficult to explain. The “market-oriented” primary care reforms in Stockholm County (2008)—free establishment of healthcare facilities by private actors and patients’ freedom to choose providers [22]— may have led to increased MHC use in lower-income groups. Although not specific to MHC use, a report on these reforms in Stockholm County found that given similar needs, utilization increased more for less affluent individuals [24], while another study reported smaller increases in General Practitioner (GP) visits for individuals with greater needs such as those with poor self-reported mental health status [25]. In a different county, Skåne County, overall utilization increased slightly more for affluent individuals [26].

Alongside primary care reforms, Region Stockholm changed its reimbursement system from majority capitation-based to majority fee-for-service (2008–2015), removing socioeconomic status from the need-adjustments [23, 74]. This change was criticized for potentially disadvantaging providers (and patients) in areas with greater needs, as they would not be compensated for longer visits, and that providers would prefer shorter visits with higher patient turnover [23]. Hence, the observed increase may be an effect of an increase in the number of shorter visits, but we cannot confirm this given the poor record coverage of primary care before 2014. However, we observed a decline in the average number of outpatient visits between 2006/2007 and 2014/2015, particularly among lower-income groups. This suggests that the reforms may have primarily benefited patients in better health or with milder mental health problems. This aligns with findings by Agerholm et al. [25], who found a smaller increase in total GP visits (2011 vs. 2007) among individuals with greater healthcare needs indicated by poor mental health, long-term limiting illness, or poor general health status [25].

The shift towards a more “pro-rich” MHC use pattern during the Covid-19 pandemic, especially among non-Nordic migrants, is significant. Compared with 2014/2015 levels, MHC use decreased for the lowest-income group but increased for other groups in 2021/2022. It is possible that certain groups delayed or avoided seeking MHC due to illness from Covid-19 or fear of infection. A study from the USA found that individuals with physical or mental health problems were more likely to delay seeking care due to the pandemic [43]. Additionally, restrictions on in-office services (physical visits) and the transition to online (digital) services may have impacted MHC access differently across socioeconomic groups. This is significant since online services were meant to enhance access. A Swedish study (pre-pandemic data from 2018) investigated the use of online primary care services for infectious conditions and found a generally “pro-rich” pattern, with higher usage among highly educated, younger individuals, urban residents, and native-born individuals [47]. In our study, we observed that the use of online MHC services increased for each income group in 2021/2022 compared with 2014/2015, with larger increases in higher-income groups compared with the lowest income group.

Another factor behind the decline in MHC use among less affluent groups in 2021/2022 could be long wait times, as indicated by falling trends in timely access to specialized services [75], and a decrease in the density of mental healthcare providers (217 per 100,000 adults in 2019 to 215 in 2022 [29, 30]). Lower socioeconomic groups are more affected by service limitations, as a pre-pandemic report showed that highly educated individuals and those with private insurance experienced shorter wait times and better healthcare [31].

Another possible reason for the declining “pro-poor” inequities could be changes in the sample composition over the years. For instance, this might occur if migrants, known to underutilize MHC services [13, 14], are overrepresented in the lowest income group. However, this impact is limited, as a decline in “pro-poor” inequities was also observed among Swedish-born individuals during Covid-19.

In addition to the general disparities faced by (non-Nordic) migrants in Sweden [13, 14], we found income-related disparities within this migrant group, specifically those born outside Europe. Lower-income migrants used less MHC services than their higher-income peers, despite having similar needs. These disparities, especially during Covid-19, could be linked to factors such as illness from Covid-19 (which disproportionately affected lower-income migrants in Stockholm [76]), avoidance of healthcare services due to fear of infection, and lack of awareness of available (online) services [77, 78].

#### Need indicators and measuring inequities

Measuring inequities relies on the quality and comprehensiveness of need indicators. Need-predicted MHC use estimates how much MHC an individual is expected to use, on average, given how individuals with the same level of need are treated by the system. This assumption therefore relies on the quality of the need indicators and is also affected by systematic variations in how people perceive their health, for example if some groups underreport/overreport their health status [72]. Psychological distress, the main indicator of need in this study, predicts MHC use well [79, 80] but does not capture all MHC needs. Including other need indicators reduced most “pro-poor” inequities. Moreover, higher utilization among lower-income persons seemed to be driven by their prior MHC use (before survey response), reflecting ongoing treatment needs for follow-up visits/refills as recommended by providers. This should be interpreted with caution due to reduced statistical power and potential bias from excluding previous users, whose mean income was slightly lower than for the rest. Combining self-reported symptoms, impaired functionality, and provider-assessments could better indicate MHC needs.

### Strengths and limitations

The linkage of surveys and registry data over four measurement points is a strength of this study, allowing for analysis of trends in horizontal equity in MHC use. The sample size per measurement point was large and allowed for period-stratified analyses.

However, the lower coverage of primary care utilization in the earlier study period could be an issue since majority of people receive MHC in primary care [81]. Our sensitivity analysis with only complete datasets yielded similar estimates and trends (likely because a significant amount of primary care utilization was still captured through psychotropic medication prescriptions). In addition, we lacked data on needs and utilization for the period 2018/2019 making it impossible to know when the shift started towards “pro-rich” MHC utilization.

Although the GHQ-12 and Kessler-6 scores were linked, their documented differences including differences in the reference period (past few weeks versus past one month) could bias inequity comparisons. Apparent reductions in inequities may be due to changes in need-measurement rather than other factors. However, sensitivity analyses and robustness checks indicated that while the change in measurement affected the inequity assessment, it did not alter the overall trends.

Another potential limitation is that the response rates have been falling over time (below 50% in the recent measurements) and this means that we do not capture inequities experienced by the most vulnerable in society who are less likely to respond to surveys. Importantly, falling response rates could introduce bias in comparing inequity over time; for instance, if the larger “pro-poor” inequities in 2014/2015 were driven by selection bias, with lower-income participants (also) being heavy users of MHC.

Lastly, we were not able to examine differences in the quality of MHC received which could have different income-related patterns. Our assumption is that each visit represents a healthcare interaction, but the content of the visit or services received could differ between income groups.

### Future research

Studies using more robust need indicators and examining differences in quality should be conducted to validate our results. It is also important to investigate whether the “pro-rich” utilization patterns observed in 2021/2022 remain prevalent in the years following the pandemic, particularly among less affluent non-Nordic migrants. Otherwise, interventions to improve MHC access, such as the TINA project (*Tidiga insatser för nyanlända* “early interventions for new migrants”), which aims to improve MHC-seeking among migrant children and adolescents [82], could be adapted and tested for adults.

## Conclusions

From 2006 to 2015, we observed that lower-income groups had increasingly higher utilization of MHC services compared with higher-income groups with the same level of self-reported psychological distress. However, during the pandemic (2021/2022), potential access problems led to diminishing of “pro-poor” inequities in the total sample, and to “pro-rich” inequities among non-Nordic migrants. This could be related to the pandemic’s negative impact on care-seeking in less affluent groups, as well as organizational changes—such as the restriction of physical visits and the transition to online healthcare services during the pandemic—which may have favored affluent individuals more than others. Unforeseeable critical events impacting on health services, such as during pandemics, may disproportionately disrupt MHC access of less affluent individuals, even when such disparity was not present before the event.

## Electronic supplementary material

Below is the link to the electronic supplementary material.

Supplementary Material 1

## Data Availability

The data that support the findings of this study are available from Region Stockholm but restrictions apply to the availability of these data, which were used under license for the current study, and so are not publicly available. Data are however available from the authors upon reasonable request and with permission of Region Stockholm. To request SPHC data, visit: https://www.ces.regionstockholm.se/projekt-och-uppdrag/halsa-stockholm/SPHC-data/.
