# Supplementary material for: More or less equal? Trends in horizontal equity in mental health care utilization in Stockholm county, Sweden (2006–2022). Repeated survey-registry linked studies
Source: Int J Equity Health. 2025 Apr 8;24:98. doi: 10.1186/s12939-025-02453-y (PMC11980088; doi:10.1186/s12939-025-02453-y)
Supplement: Supplementary file 1 — Supplementary Material 1 [file 12939_2025_2453_MOESM1_ESM.docx]

**More or Less Equal? Trends in Horizontal Equity in Mental Health Care Utilization in Stockholm County, Sweden (2006 – 2022).** *Repeated survey-registry linked studies*

Joseph Junior Muwonge^1,2^ (ORCID: 0000-0002-9219-9752), Beata Jablonska^1,2^ (ORCID: 0000-0002-0246-6643), Christina Dalman^1,2^ (ORCID: 0000-0002-3579-2357), Bo Burström^1,2^ (ORCID: [0000-0001-5770-9422](https://orcid.org/0000-0001-5770-9422)), Maria Rosaria Galanti^1^ (ORCID: 0000-0002-7805-280X), Anna-Clara Hollander^1^ (ORCID:[0000-0002-1246-5804](https://orcid.org/0000-0002-1246-5804))

1. Department of Global Public Health, Karolinska Institute, Stockholm, Sweden
2. Centre for Epidemiology and Community Medicine, Stockholm Health Care Services, Region Stockholm, Stockholm, Sweden

Corresponding author

Name: Joseph Junior Muwonge

Contact: [joseph.junior.muwonge@ki.se](mailto:joseph.junior.muwonge@ki.se)

**Methods**

Measurement of Mental Health Care use

- Psychiatric diagnoses were based on the tenth revision of the International Classification of Diseases (ICD-10) diagnostic codes: F00-F99, X60-X84, Z72820, Z915, G47, R45851, and T1491.
- Psychotropic medication was identified using the following Anatomical Therapeutic Chemical code (ATC) codes: N05A, N05B, N05C, N06A, N06B, N07BB, and N07BC
- Psychosocial support in primary and secondary outpatient care was identified using the following codes: Uppdragtyp (301, 320), KLIN (950, 951, 955), and VDG1-5 (74, 75, 96; meeting with a psychologist, counsellor, and psychotherapist).

*
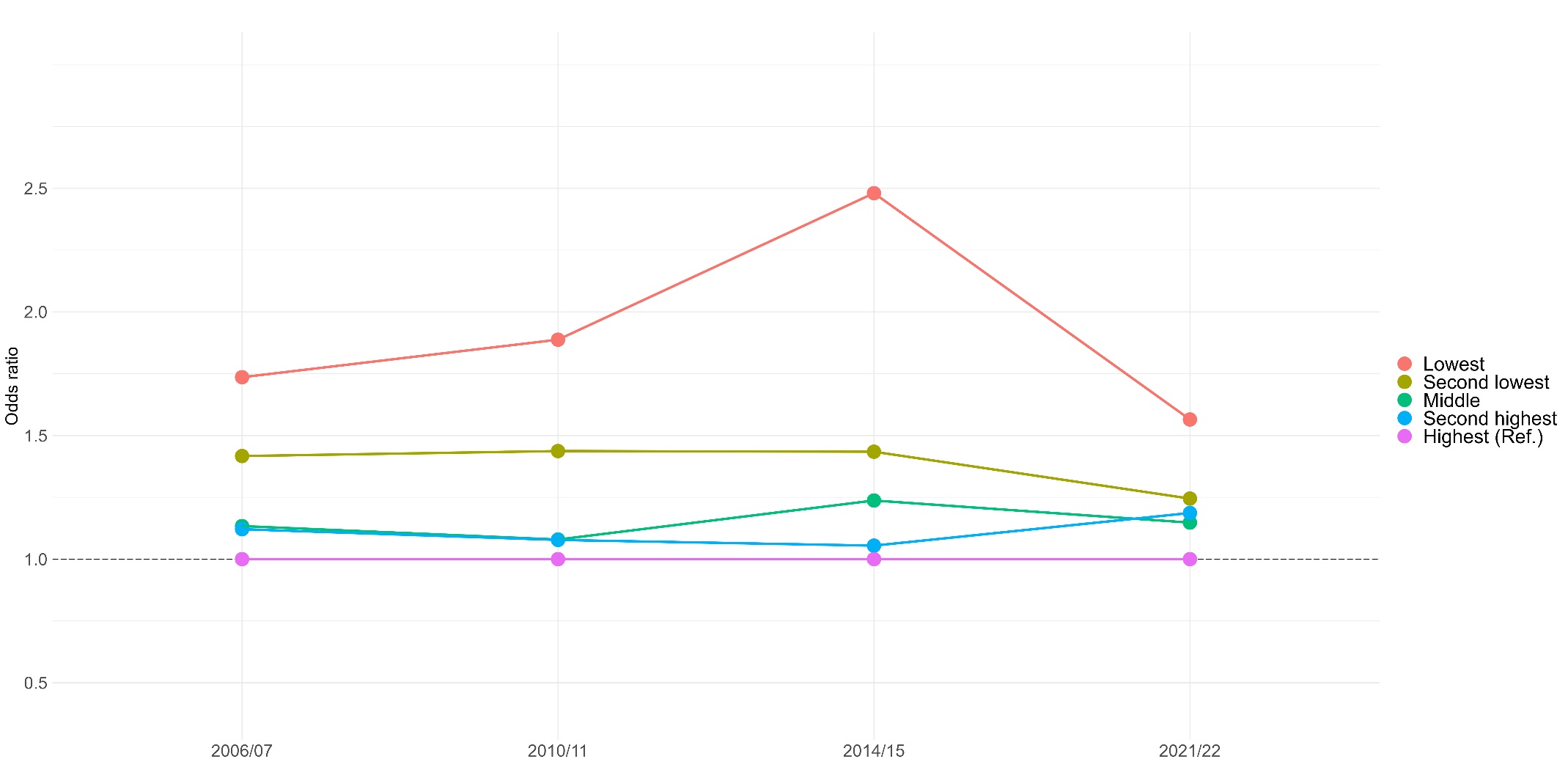
*

**Figure S1. Trends in income-related differences in MHC access using logistic regressions.** *Odds ratios for the association between household income and MHC access. Model adjusted for age-group, sex, migration status, and psychological distress. 95% confidence limits not shown for simplicity (see table S1 for 95% CL).*

*
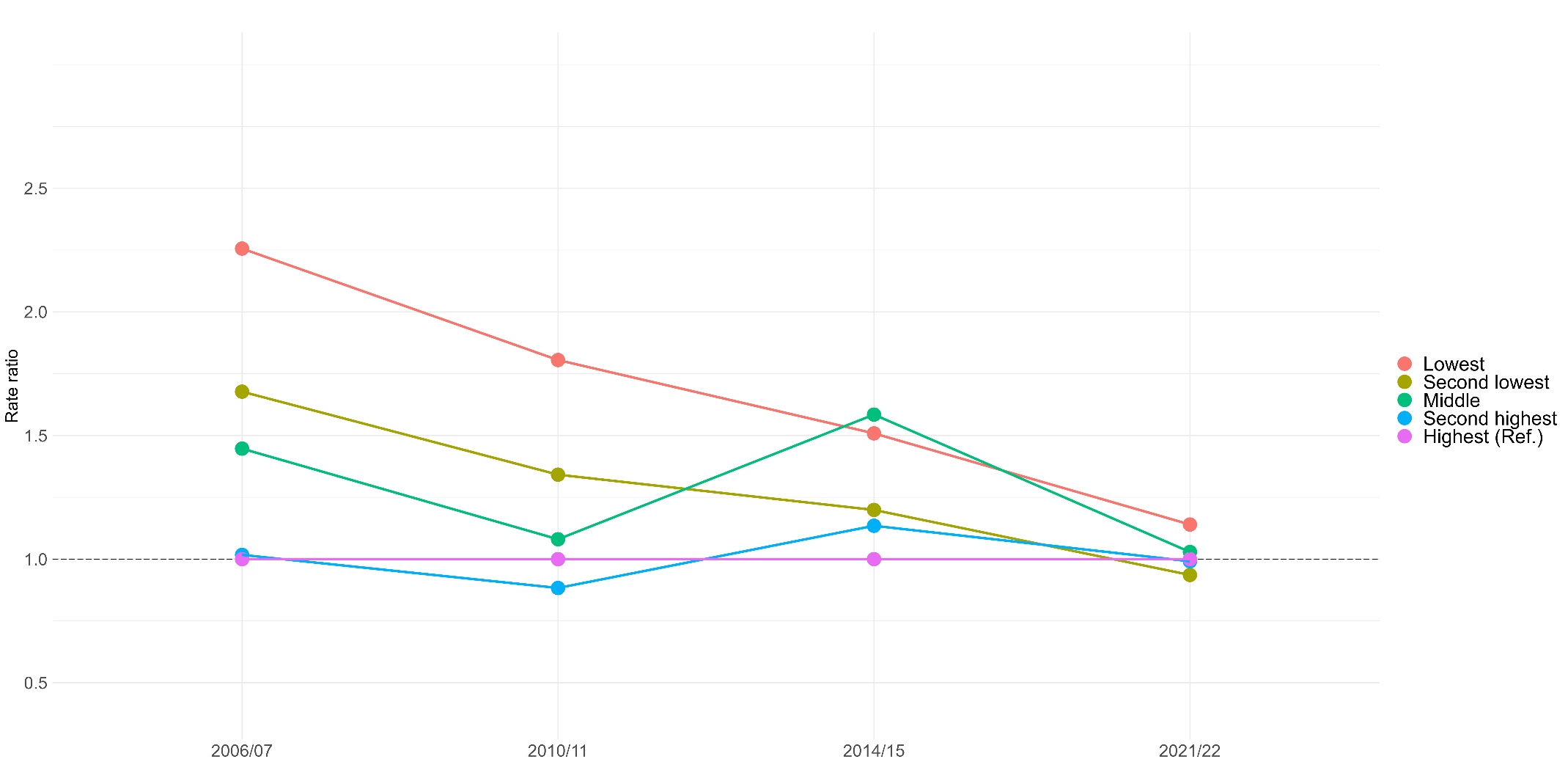
*

**Figure S2. Trends in income-related differences in the frequency of outpatient visits using zero-truncated negative binomial regressions.** *Rate ratios for the association between household income and number of outpatient visits. Model adjusted for age-group, sex, migration status, and psychological distress. 95% confidence limits not shown for simplicity (see table S2 for 95% CL).*

**Table S1.** **Trends in income-related differences in MHC access using logistic regressions.** *Odds ratios for the association between household income and MHC access.*

|  | 2006/07 | 2010/11 | 2014/15 | 2021/22 |
| --- | --- | --- | --- | --- |
|  | *OR (95% CL)* | *OR (95% CL)* | *OR (95% CL)* | *OR (95% CL)* |
| Lowest | **1.74 (1.51, 2.00)** | **1.89 (1.63, 2.19)** | **2.48 (2.09, 2.95)** | **1.56 (1.33, 1.84)** |
| Second lowest | **1.42 (1.24, 1.62)** | **1.44 (1.24, 1.67)** | **1.43 (1.21, 1.70)** | **1.24 (1.06, 1.46)** |
| Middle | 1.13 (0.99, 1.30) | 1.08 (0.93, 1.25) | **1.24 (1.04, 1.48)** | 1.15 (0.97, 1.35) |
| Second highest | 1.12 (0.98, 1.29) | 1.08 (0.92, 1.26) | 1.05 (0.88, 1.27) | **1.19 (1.01, 1.40)** |
| Highest (ref.) |  |  |  |  |
|  | - *Logistic regression used and model adjusted for age-group, sex, migration status, and psychological distress* - *Survey weights and strata (svyset procedure in stata) used.* - ***Bold*** *font indicates statistical significance.* | | | |

**Table S2. Trends in income-related differences in the frequency of outpatient visits using zero-truncated negative binomial regressions.** *Rate ratios for the association between household income and number of outpatient visits.*

|  | **2006/07** | **2010/11** | **2014/15** | **2021/22** |
| --- | --- | --- | --- | --- |
|  | *RR (95% CL)* | *RR (95% CL)* | *RR (95% CL)* | *RR (95% CL)* |
| Low income | **2.26 (1.48, 3.43)** | **1.81 (1.29, 2.53)** | **1.51 (1.08, 2.10)** | 1.14 (0.82, 1.58) |
| 2 | **1.68 (1.09, 2.57)** | 1.34 (0.95, 1.90) | 1.20 (0.86, 1.67) | 0.94 (0.67, 1.31) |
| 3 | 1.45 (0.91, 2.29) | 1.08 (0.71, 1.64) | **1.58 (1.04, 2.41)** | 1.03 (0.73, 1.45) |
| 4 | 1.02 (0.63, 1.64) | 0.88 (0.58, 1.34) | 1.13 (0.78, 1.66) | 0.99 (0.69, 1.42) |
| High income (ref.) |  |  |  |  |
|  | - *Zero-truncated negative binomial regression used, and model adjusted for age-group, sex, migration status, and psychological distress* - *Survey weights and strata (svyset procedure in stata) used.* - ***Bold*** *font indicates statistical significance.* | | | |

**Figure S3. Concentration Curve showing the income-related inequalities in the probability of poor/very poor general health status.** *CI = Concentration index, *** - P-value <0.0001. Curves above the Line of Equality indicate higher concentration of poor/very poor general health status in individuals with lower income. The closer the curve is to the Line of Equality, the more equal the distribution of poor/very poor general health status among individuals with varying income.*

**Figure S4. Concentration Curve showing the income-related inequalities in the probability of having long-term limiting illness or health problems.** *CI = Concentration index,* **** - P-value <0.0001. Curves above the Line of Equality indicate higher concentration of long-term illness in individuals with lower income. The closer the curve is to the Line of Equality, the more equal the distribution of long-term illness among individuals with varying income.*

**Table S3. Stratified analyses of income related inequities in MHC use**

| **Level** | **2006/07** | | **2010/11** | | **2014/15** | | **2021/22** | |
| --- | --- | --- | --- | --- | --- | --- | --- | --- |
|  | **Model 1** | **Model 2** | **Model 1** | **Model 2** | **Model 1** | **Model 2** | **Model 1** | **Model 2** |
|  | *HI (95% CL)* | *HI (95% CL)* | *HI (95% CL)* | *HI (95% CL)* | *HI (95% CL)* | *HI (95% CL)* | *HI (95% CL)* | *HI (95% CL)* |
| **Overall** | **-0.057 (-0.079, -0.034)** | 0.000 (-0.023, 0.022) | **-0.081 (-0.105, -0.056)** | -0.022 (-0.046, 0.002) | **-0.130 (-0.159, -0.102)** | **-0.078 (-0.106, -0.049)** | **-0.034 (-0.06, -0.009)** | -0.010 (-0.035, 0.015) |
| **Sex** |  |  |  |  |  |  |  |  |
| *Men* | **-0.102 (-0.141, -0.063)** | -0.036 (-0.074, 0.002) | **-0.096 (-0.139, -0.053)** | -0.035 (-0.078, 0.008) | **-0.155 (-0.204, -0.106)** | **-0.102 (-0.153, -0.052)** | -0.009 (-0.052, 0.034) | 0.015 (-0.028, 0.057) |
| *Women* | -0.011 (-0.039, 0.017) | **0.040 (0.011, 0.068)** | **-0.057 (-0.087, -0.026)** | 0.001 (-0.029, 0.030) | **-0.106 (-0.141, -0.072)** | **-0.053 (-0.088, -0.018)** | **-0.039 (-0.071, -0.008)** | -0.014 (-0.045, 0.018) |
| **Age** |  |  |  |  |  |  |  |  |
| *18-29* | **-0.077 (-0.139, -0.014)** | -0.020 (-0.081, 0.041) | **-0.070 (-0.131, -0.009)** | -0.037 (-0.097, 0.023) | **-0.161 (-0.227, -0.094)** | **-0.125 (-0.192, -0.058)** | -0.048 (-0.108, 0.011) | -0.032 (-0.090, 0.025) |
| *30-64* | **-0.093 (-0.117, -0.068)** | -0.019 (-0.043, 0.006) | **-0.111 (-0.138, -0.084)** | **-0.031 (-0.057, -0.004)** | **-0.135 (-0.167, -0.104)** | **-0.069 (-0.101, -0.038)** | **-0.041 (-0.069, -0.013)** | -0.007 (-0.034, 0.021) |
| **Migration status** |  |  |  |  |  |  |  |  |
| *Nordic region (incl. Sweden)* | **-0.092 (-0.117, -0.066)** | **-0.040 (-0.064, -0.015)** | **-0.115 (-0.142, -0.088)** | **-0.056 (-0.082, -0.030)** | **-0.178 (-0.210 ,-0.147)** | **-0.119 (-0.150, -0.088)** | **-0.101 (-0.129 ,-0.074)** | **-0.074 (-0.101, -0.047)** |
| *Only Sweden* | **-0.087 (-0.113, -0.061)** | **-0.038 (-0.063, -0.013)** | **-0.112 (-0.140, -0.084)** | **-0.058 (-0.085, -0.031)** | **-0.172 (-0.204, -0.14)** | **-0.112 (-0.144, -0.080)** | **-0.102 (-0.130, -0.074)** | **-0.075 (-0.103, -0.048)** |
| *Non-Nordic migrants* | **0.067 (0.012, 0.121)** | **0.094 (0.041, 0.147)** | -0.004 (-0.066, 0.058) | 0.026 (-0.034, 0.086) | -0.035 (-0.102 ,0.031) | -0.011 (-0.079, 0.057) | 0.057 (-0.005 ,0.120) | **0.082 (0.020, 0.144)** |
| *Only non-European* | 0.034 (-0.035, 0.104) | **0.076 (0.009, 0.144)** | 0.006 (-0.072, 0.083) | 0.035 (-0.040, 0.111) | -0.052 (-0.135, 0.031) | -0.034 (-0.119, 0.052) | **0.100 (0.024, 0.176)** | **0.117 (0.041, 0.193)** |
|  | - *Model 1 – standardized for self-reported psychological distress* - *Model 2 - standardized for self-reported psychological distress, general health status, long-term limiting illness* - ***Bold*** *font indicates statistical significance.* | | | | | | | |

**Table S4. Sensitivity analyses – Income-related inequities in MHC care use at least once**

|  | **2006/2007** | **2010/2011** | **2014/2015** | **2021/2022** |
| --- | --- | --- | --- | --- |
|  | *HI (95% CL)* | *HI (95% CL)* | *HI (95% CL)* | *HI (95% CL)* |
| Only complete health records used (no primary or private care) | **-0.048 (-0.071 ,-0.024)** | **-0.075 (-0.102 ,-0.049)** | **-0.129 (-0.159 ,-0.099)** | **-0.037 (-0.065 ,-0.010)** |
| Incident MHC use ”removing previous users” | -0.032 (-0.070, 0.007) | -0.037 (-0.078, 0.004) | **-0.065 (-0.120, -0.011)** | 0.027 (-0.022, 0.075) |
|  | - *Standardized for self-reported psychological distress* - ***Bold*** *font indicates statistical significance.* | | | |

**Table S5. Stratified analyses of income related inequities in the frequency of primary and secondary outpatient visits, conditional on having at least one outpatient visit.**

| **Level** | **2006/07** | | **2010/11** | | **2014/15** | | **2021/22** | |
| --- | --- | --- | --- | --- | --- | --- | --- | --- |
|  | **Model 1** | **Model 2** | **Model 1** | **Model 2** | **Model 1** | **Model 2** | **Model 1** | **Model 2** |
|  | *HI (95% CL)* | *HI (95% CL)* | *HI (95% CL)* | *HI (95% CL)* | *HI (95% CL)* | *HI (95% CL)* | *HI (95% CL)* | *HI (95% CL)* |
| **Overall** | **-0.108 (-0.153, -0.063)** | **-0.106 (-0.151, -0.061)** | **-0.105 (-0.160, -0.050)** | **-0.103 (-0.159, -0.048)** | **-0.051 (-0.096, -0.006)** | -0.048 (-0.095, 0.000) | **-0.039 (-0.074, -0.004)** | -0.033 (-0.068, 0.002) |
| **Sex** |  |  |  |  |  |  |  |  |
| *Men* | **-0.113 (-0.191, -0.035)** | **-0.109 (-0.188, -0.031)** | **-0.084 (-0.175, 0.007)** | **-0.093 (-0.184, -0.001)** | -0.010 (-0.078, 0.058) | -0.015 (-0.087, 0.058) | -0.024 (-0.078, 0.031) | -0.017 (-0.071, 0.037) |
| *Women* | **-0.101 (-0.156, -0.047)** | **-0.101 (-0.156, -0.047)** | **-0.115 (-0.183, -0.046)** | **-0.098 (-0.165, -0.031)** | **-0.073 (-0.130, -0.015)** | **-0.065 (-0.126, -0.004)** | **-0.045 (-0.089, -0.001)** | -0.036 (-0.081, 0.008) |
| **Age** |  |  |  |  |  |  |  |  |
| *18-29* | -0.054 (-0.142, 0.035) | -0.065 (-0.151, 0.021) | **-** | **-** | **-0.106 (-0.192, -0.020)** | **-** | -0.027 (-0.098, 0.043) | - |
| *30-64* | **-0.122 (-0.175, -0.070)** | **-0.118 (-0.172, -0.065)** | **-0.108 (-0.176, -0.041)** | -0.027 (-0.083, 0.030) | -0.032 (-0.084, 0.021) | -0.027 (-0.083, 0.030) | **-0.042 (-0.081, -0.004)** | -0.034 (-0.071, 0.004) |
| **Migration status** |  |  |  |  |  |  |  |  |
| *Nordic region (incl. Sweden)* | **-0.129 (-0.178, -0.079)** | **-0.128 (-0.177, -0.078)** | **-0.135 (-0.197, -0.073)** | **-0.125 (-0.187, -0.063)** | **-0.086 (-0.137, -0.034)** | **-0.077 (-0.131, -0.023)** | **-0.064 (-0.101, -0.026)** | **-0.055 (-0.091, -0.018)** |
| *Non-Nordic migrants* | - | - | - | - | - | - | - | - |
|  | - *Model 1 – standardized for self-reported psychological distress* - *Model 2 – standardized for self-reported psychological distress, general health status, long-term limiting illness* - *(-) model did not converge due to few cases.* - ***Bold*** *font indicates statistical significance.* | | | | | | | |

**Table S6. Trends in proportions of MHC use 6 months after survey response by level or type of service, unweighted proportions.**

|  | **2006/2007** | **2010/2011** | **2014/2015** | **2021/2022** |
| --- | --- | --- | --- | --- |
| Psychotropic medication | 11.2% | 11.4% | 13.0% | 14.3% |
| Inpatient | 0.35% | 0.37% | 0.32% | 0.43% |
| Outpatient - total | 4.81% | 5.90% | 9.99% | 13.02% |
| Primary care | 1.06% | 1.81% | 5.64% | 8.86% |
| Secondary outpatient care | 3.86% | 4.30% | 5.26% | 5.78% |
| Online services | 0.98% | 1.2% | 1.08% | 7.06% |
| Physical services | 4.59% | 5.7% | 9.81% | 10.37% |

***Note - Sharp increases over time in primary care are more likely due to improved reporting than a real increase in service utilization.***

**Table S7. Need-standardized probabilities in MHC use by healthcare level and type of contact in 2014/2015 and 2021/2022.** *Need proxied by psychological distress*

|  | **Primary care** | | **Specialist outpatient** | | **Digital contact** | | **Physical contact** | | **Psychotropic medication** | |
| --- | --- | --- | --- | --- | --- | --- | --- | --- | --- | --- |
|  | **2014/2015** | **2021/2022** | **2014/2015** | **2021/2022** | **2014/2015** | **2021/2022** | **2014/2015** | **2021/2022** | **2014/2015** | **2021/2022** |
| **Household income**, quintiles |  |  |  |  |  |  |  |  |  |  |
| Low | 6.9% | 8.9% | 10.3% | 9.1% | 2.0% | 8.3% | 15.5% | 13.7% | 16.6% | 14.2% |
| 2 | 5.9% | 8.9% | 5.7% | 5.4% | 1.2% | 7.4% | 10.6% | 9.7% | 11.9% | 13.1% |
| 3 | 5.3% | 8.7% | 3.7% | 5.6% | 0.9% | 7.4% | 8.2% | 10.0% | 11.4% | 13.1% |
| 4 | 4.7% | 8.9% | 3.3% | 4.6% | 0.6% | 6.8% | 7.4% | 9.4% | 10.6% | 13.9% |
| High | 4.2% | 7.3% | 3.1% | 4.3% | 0.7% | 5.4% | 6.5% | 8.3% | 10.4% | 12.5% |
| HI | **-0.104 (-0.149, -0.059)** | -0.032 (-0.067, 0.004) | **-0.280 (-0.323, -0.236)** | **-0.178 (-0.22, -0.136)** | **-0.259 (-0.355, -0.162)*** | **-0.089 (-0.128, -0.050)** | **-0.204 (-0.237, -0.170)** | **-0.111 (-0.144, -0.079)** | **-0.113 (-0.145, -0.082)** | -0.024 (-0.053, 0.006) |
|  | ****Imprecise/unreliable estimate due to low statistical power since only 1.08% used online (digital) services in 2014/2015.*** | | | | | | | | | |

**Table S8. Sensitivity analyses – Testing robustness of trend results due to change in distress measurement in 2021**

|  | **2006/2007** | **2010/2011** | **2014/2015** | **2021/2022** |
| --- | --- | --- | --- | --- |
|  | *HI (95% CL)* | *HI (95% CL)* | *HI (95% CL)* | *HI (95% CL)* |
| Need-standardized using only psychological distress – main model | **-0.057 (-0.079, -0.034)** | **-0.081 (-0.105, -0.056)** | **-0.130 (-0.159, -0.102)** | **-0.034 (-0.06, -0.009)** |
| Need-standardized using general health status and long-term limiting illness | -0.014 (-0.036, 0.009) | **-0.032 (-0.057, -0.008)** | **-0.082 (-0.111, -0.053)** | **-0.043 (-0.068, -0.018)** |
| Only general health status | -0.010 (-0.033, 0.012) | **-0.032 (-0.056, -0.007)** | **-0.083 (-0.113, -0.054)** | **-0.047 (-0.072, -0.023)** |
|  | - *Standardized for self-reported psychological distress* - ***Bold*** *font indicates statistical significance.* | | | |

**Table S9. Sensitivity analyses – Testing robustness of trend results in frequency of visits by removing primary care visits**

| **Level** | **2006/07** | | **2010/11** | | **2014/15** | | **2021/22** | |
| --- | --- | --- | --- | --- | --- | --- | --- | --- |
|  | **Model 1** | **Model 2** | **Model 1** | **Model 2** | **Model 1** | **Model 2** | **Model 1** | **Model 2** |
|  | *HI (95% CL)* | *HI (95% CL)* | *HI (95% CL)* | *HI (95% CL)* | *HI (95% CL)* | *HI (95% CL)* | *HI (95% CL)* | *HI (95% CL)* |
| All outpatient visits (incl. Primary care) | **-0.108 (-0.153, -0.063)** | **-0.106 (-0.151, -0.061)** | **-0.105 (-0.160, -0.050)** | **-0.103 (-0.159, -0.048)** | **-0.051 (-0.096, -0.006)** | -0.048 (-0.095, 0.000) | **-0.039 (-0.074, -0.004)** | -0.033 (-0.068, 0.002) |
| Specialized outpatient visits | **-0.109 (-0.158, -0.060)** | **-0.108 (-0.158, -0.059)** | **-0.114 (-0.178, -0.050)** | **-0.118 (-0.181, -0.054)** | -0.027 (-0.080, 0.026) | -0.025 (-0.081, 0.030) | -0.032 (-0.080, 0.016) | -0.027 (-0.075, 0.021) |
